# Photocatalytic Reduction of Cr(VI) in the Presence of Humic Acid Using Immobilized Ce–ZrO_2_ under Visible Light

**DOI:** 10.3390/nano10040779

**Published:** 2020-04-18

**Authors:** Fabrício Eduardo Bortot Coelho, Victor M. Candelario, Estêvão Magno Rodrigues Araújo, Tânia Lúcia Santos Miranda, Giuliana Magnacca

**Affiliations:** 1 Department of Chemistry, University of Torino, Via P.Giuria 7, 10125 Torino, Italy; fabricioeduardo.bortotcoelho@unito.it; 2 LiqTech International A/S, Industriparken 22 C, 2750 Ballerup, Denmark; vcl@liqtech.com; 3 Chemical Engineering Department, Federal University of Minas Gerais, Av.Antônio Carlos 6627, 30000-000 Belo Horizonte, Brazil; estevaomagno@gmail.com (E.M.R.A.); tania@deq.ufmg.br (T.L.S.M.)

**Keywords:** hexavalent chromium, photocatalysis, zirconia, humic acid, photoreduction, zinc, natural organic matter, catalyst immobilization

## Abstract

Cr(VI) has several industrial applications but it is one of the most dangerous pollutants because of its carcinogenicity and high toxicity. Thus, the removal of Cr(VI) by photocatalytic reduction was investigated. The catalyst applied, Ce–ZrO_2_, was immobilized, through a sol–gel process on a silicon carbide (SiC) support, to increase the efficiency and avoid using suspended nanoparticles. The influence of initial pH, humic acid (HA), and catalyst dosage was investigated for Cr(VI) containing solutions. Then, a real galvanizing industry effluent (Cr(VI) = 77 mg L^-1^mg.L^−1^, Zn = 1789 mg L^−1^) was treated. It was observed that Cr(VI) adsorption and photoreduction are greatly favored at low pH values. HA can decrease Cr(VI) adsorption but also acts as holes scavenger, reducing the electron–hole recombination, favoring then the photoreduction. With the immobilized Ce–ZrO_2_, more than 97% of Cr(VI) was removed from the diluted effluent. These results indicate the feasibility to treat Cr(VI) effluents even in the presence of other metals and natural organic matter. The developed material has great chemical and mechanical resistances and avoids the use of nanoparticles, dangerous for the environment and hard to recover. Moreover, solar light can be used to drive the process, which contributes to the development of more sustainable, cleaner, and cost-effective wastewater treatments.

## 1. Introduction

Hexavalent chromium is widely used in several industrial processes, such as metal plating, leather tanning, pigment, and refractory production [1,2,3]. There is a serious global concern about the highly toxic effluents generated in these processes, since several cases of chromium contamination have been reported in soil, ground, and surface waters caused by the leakage from landfill sites or inappropriate treatment methods [4]. In aquatic environments, chromium primarily occurs in its hexavalent—Cr(VI)—and trivalent—Cr(III)—states. Hexavalent chromium is considered one of the most dangerous pollutants due to its high toxicity to humans, animals, and plants. It is widely recognized for its teratogenic and carcinogenic effects on human health [5,6]. In addition, high concentrations of Cr(VI) can inhibit significantly the biomass growth during wastewater biological treatments [7]. 

Several methods can be applied to treat Cr(VI) effluents, such as chemical precipitation, ion-exchange, filtration, solvent extraction, electrochemical, and biological processes [8,9]. Adsorption has been widely studied [10,11] but the large volume of sludge residue and the expensive and complex materials used as adsorption phase are still major drawbacks of this method that must be overcome [9,12]. Since Cr(III) is about 500 times less toxic than its hexavalent form [13] and it can usually be immobilized through precipitation or adsorption onto a solid phase [4], several Cr(VI) treatment processes start with the reduction of this metal to its trivalent form. However, this reduction is not easily achieved due to the higher Cr(VI) stability in terms of the reduction potential [14], whereas the chemical reduction is an expensive process with a large footprint [15].

In this context, the photocatalytic reduction of Cr(VI) is attracting interest for being more practical and cleaner [15], especially considering the possibility of using solar light to drive the process, which could reduce considerably the costs. Among the photocatalysts available, semiconductors can be used since their conduction band (CB) is more negative than the reduction potential of Cr(VI) (i.e., ~0.36 at pH seven) [15]. However, as the reduction potential of Cr(VI) becomes more negative at higher pH, the photocatalytic reduction of the Cr(VI) ion is favored in a lower pH range. Oxides such as TiO_2_ [14,16,17] and ZnO [2] have been reported as efficient photocatalysts for this process but these oxides may not be totally stable in acid media [18,19]. 

Therefore, an alternative is to use zirconium dioxide (ZrO_2_) as a photocatalyst since this oxide has outstanding chemical [20], thermal, and mechanical stability [21]. In addition, zirconia is a powerful candidate for Cr(VI) photocatalytic reduction, because the lowest potential of the CB is −1.0 eV (vs. normal hydrogen electrode (NHE), pH 0), much more negative than that of TiO_2_ anatase (−0.1 eV), whereas the highest potential of the valence band (VB) is +4.0 eV, more positive than that of TiO_2_ (+3.1 eV) [22]. On the other hand, these band energy levels imply a large bandgap value (ca. 5.0 eV), which does not allow the activation of zirconia with solar light. Therefore, our group developed a nanosized Ce-doped zirconia (Ce–ZrO_2_) capable of generating photo-induced electrons and holes under visible light irradiation [22]. The cerium doping adds Ce 4f empty intraband states, which act as a bridge between the VB and the CB of zirconia. In a “double jump” mechanism, low-energy photons (visible light) is absorbed, leading to the excitation of electrons from the valence band to the conduction band [22,23]. This material found applications in degrading pollutants [23,24,25] and promoting water splitting [26].

The use of suspended photocatalysts, especially nanosized ones, presents a major disadvantage related to the catalyst recovery. At the end of the treatment, in fact, a time-consuming and expensive solid/liquid separation step is required to recycle the catalyst and avoid its leakage to the environment, since nanomaterials can harm human health [27] and have an ecotoxicological impact [28]. In addition, suspended catalyst particles have a strong light absorption that decreases the depth of penetration of the light [29]. Therefore, a great effort has been made to immobilize photocatalysts on supports in order to obtain better light absorption efficiency and eliminate the catalyst recovery step [30,31]. 

Regarding the presence of natural organic matter (NOM) such as humic (HA) and fulvic (FA) acids in surface and wastewaters, its effects on Cr(VI) toxicity and treatment are still unclear. Dissolved NOM can promote the Cr(VI) reduction [32,33] but also increases the solubility and mobility of Cr(VI) species in soils [16,33,34]. In addition, undissolved NOM in soils can immobilize Cr(VI) via adsorption [35]; however, the solid–liquid separation in the Cr removal process can be hindered by soluble Cr(III)–HA complexes [36]. 

Previous works studied the use of zirconia alone (e.g., N–ZrO_2_ [37], amorphous ZrO_2_ [38]) or in mixtures with other oxides (e.g., CuO/ZrO_2_ [39], ZrO_2_/TiO_2_[40], Fe–ZrO_2_/TiO_2_ [41,42,43]) as catalysts for the photoreduction of Cr(VI). However, there is no report on the use of cerium-doped zirconia for Cr(VI) removal nor the use of immobilized Ce–ZrO_2_ in photocatalysis. 

In this context, the objective of this work was to study the photocatalytic reduction of Cr(VI) using Ce-doped ZrO_2_ under visible light irradiation in aqueous systems. Moreover, it was evaluated how the presence of HA affects the Cr(VI) adsorption and reduction, since humic-like substances are usually present in wastewaters. Initially, in order to study the effects of some parameters (e.g., initial pH value and catalyst dosage) on Cr(VI) reduction, experiments were performed with suspended Ce–ZrO_2_ particles, using solutions containing Cr(VI). Then, Ce–ZrO_2_ immobilized on a macroporous silicon carbide (SiC) was evaluated for the photoreduction of Cr(VI) in a synthetic solution and in a galvanizing industry effluent ([Cr]_total_ = 89 mg L^−1^, [Cr(VI)] = 77 mg L^−1^, [Zn] = 1789 mg L^−1^).

## 2. Materials and Methods

### 2.1. Synthesis and Characterization of the Ce–ZrO_2_ Photocatalyst

In order to remove Cr(VI) by photoreduction, the catalyst applied was zirconia doped with 0.5% molar of cerium. This catalyst was tested as a suspended powder or immobilized on a support. In the first case, Ce–ZrO_2_ nanopowder was synthesized by a sol–gel route developed in previous work [25]. In brief, for the synthesis processes, 5 mL of zirconium propoxide Zr(OC_3_H_7_)_4_ (CAS 23519-77-9, 70 wt %, Sigma-Aldrich, St. Louis, MO, United States) were mixed with 5 mL of 2-propanol (CAS 67-63-0, 99.5%, Sigma-Aldrich, St. Louis, MO, United States). Then, 28 mg of Ce(NH_4_)_2_(NO_3_)_6_ (CAS 16774-21-3, purity >98.5%, Sigma-Aldrich, St. Louis, MO, United States), dissolved in 5 mL of distilled water, were added to the first solution to start hydrolysis. The resulting gel was kept overnight at room temperature and then dried at 80 °C. After aging at room temperature for 10 days, the xerogel was calcined in a muffle furnace at 500 °C in air for 4 h. 

A sol–gel process, followed by dip coating and sintering, were applied to immobilize Ce–ZrO_2_ on a silicon carbide support with a ZrO_2_ intermediate later, which was supplied by LiqTech International A/S (Ballerup, Denmark). A SiC support was selected owing to this material high porosity, mechanical strength, and good thermal and chemical resistance [44,45,46], which make it applicable in harsh environments, such as high temperature and acid media, where other materials (e.g., alumina and silica) fail [47,48]. The coating liquid was a colloidal sol prepared through an adapted procedure for yttria-stabilized zirconia synthesis [49]. For that, 5 mL of zirconium propoxide and 28 mg of Ce(NH_4_)_2_(NO_3_)_6_ were diluted in 25 mL of 2-propanol. Next, 60 mL of a 0.05 M solution of HNO_3_ (CAS 7697-37-2, >65%, Sigma-Aldrich, St. Louis, MO, United States) was added and the system kept under reflux conditions until obtaining a transparent gel. The support was then dip-coated with this gel and dried overnight under room temperature. Next, the coated support was calcined in a muffle furnace at 500 °C in air for 4 h. All chemicals were used as received, without further purification.

Zeta potential measurements were performed on a Zetasizer Nano ZS (Malvern Instruments, Malvern, United Kingdom) using principles of laser Doppler velocimetry and phase analysis light scattering (M3–PALS technique). Briefly, 0.1% *w/v* suspensions of nanoparticles were prepared with a NaCl 0.01 M aqueous solution and ultrasonicated for 10 min before the analysis.

Electronic microscopy images of the immobilized Ce–ZrO_2_ were obtained with the Field Emission Gun Scanning Electronic Microscope FIB–FESEM S9000G (Tescan, Brno-Kohoutovice, Czech Republic).

X-ray diffraction analyses (XRD) were performed in the diffractometer PW3040/60 X’Pert PRO MPD (Malvern Panalytical, Almelo, Netherlands), operating at 45 kV, 40 mA, with a Cu Kα radiation source (λ = 1.5418 Å) and a Bragg–Brentano geometry over the range 10° < 2θ < 80°.

Diffuse Reflectance Spectroscopy (DRS) data were recorded in the 200–700 nm range using a Cary 5000 spectrometer (Varian, Palo Alto, CA, United States), coupled with an integration sphere for diffuse reflectance studies. A sample of PTFE with 100% reflectance was used as the reference. The optical bandgap energy has been calculated from the Tauc plot.

### 2.2. Photocatalytic Experiments

Stock Cr(VI) and humic acid solutions were prepared by dissolving the desired amounts of K_2_Cr_2_O_7_ (CAS 7778-50-9, Sigma-Aldrich, >99 %) and humic acid sodium salt (CAS 68131-04-4, Sigma-Aldrich, MP > 300 °C) in distilled water. The pH values of the solutions were adjusted to the desired value using aqueous solutions of NaOH and HCl. Wastewater from a Cr(VI) bath used to passivate steel pieces coated with fused zinc was collected from a galvanizing industry in the state of Minas Gerais (Brazil). This effluent has a density of 1.007 g cm^−3^ at 20 °C, a pH value of 2.9, and a metal concentration of: [Cr]_total_ = 89 mg L^−1^, [Cr(VI)] = 77 mg L^−1^, [Zn] = 1789 mg L^−1^.

For the first part of the photocatalytic reduction experiments, with powdery Ce–ZrO_2_, a specific amount of the catalyst was added to the Cr(VI) or Cr(VI) + HA solutions in order to achieve the desired dosage. As a reactor, a 100 mL borosilicate glass was used. The pH was then adjusted to the desired value. Prior to irradiation, the mixture was kept at room temperature (25 ± 3 °C), under stirring, in the dark, for 3 h in order to achieve the adsorption equilibrium. Then, the mixture was irradiated with visible light for 3 h under stirring, without any injection of gas. Samples were collected in specific intervals of time and filtered with 0.27 µm siring filters prior to Cr(VI) analysis. All experiments were performed in duplicate.

As the light source, an 18 W white LED lamp (Wellmax, Shanghai, China) was used. This lamp has a color temperature of 6500 K and, as indicated by the manufacturer, no UV/IR emission. In order to confirm the lamp emission, the UV irradiance (λ < 400 nm) was measured with the light meter HD 2302.0 (Delta OHM, Caselle di Selvazzano, Italy). No UV emission was detected; thus, no filter was applied in the photocatalysis experiments. When the lamp was positioned just above the beaker, the measured VIS-light irradiance (400 < λ < 700 nm) was 1000 W m^−2^.

**Figure 1:**
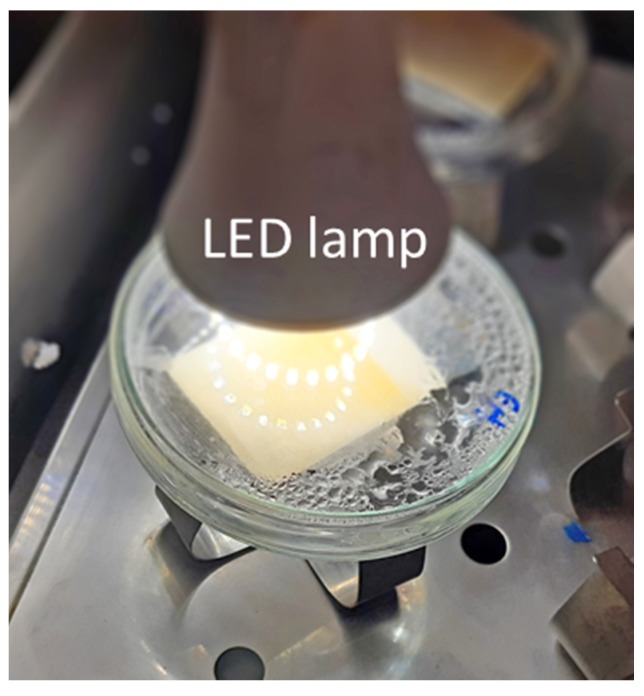
Experimental setup for Cr(VI) photocatalytic reduction with immobilized Ce–ZrO_2_.

For the second part of the experiments, Ce–ZrO_2_ immobilized on the support (SiC/ZrO_2_) was applied to the Cr(VI) photoreduction. A piece of 4 × 6 cm was added to a petri dish containing 30 mL of a Cr(VI) model solution or the galvanizing industry effluent (diluted 7× in order to achieve a Cr concentration of 11 mg L^−1^) and positioned under the above-mentioned lamp (Figure 1). In order to keep the system under stirring, the petri dish was placed in an orbital shaker at 400 rpm. A test with the diluted effluent spiked with HA was also performed. In the experiments with the Cr(VI) solution the initial pH was adjusted to 4, while for the effluent, it utilized the natural value of the diluted effluent, around 3.5. Next, the system was kept in the dark for 6 h, for allowing the adsorption equilibrium, and then irradiated by visible light for 6 h. Samples were collected at specific intervals of time and stored for analysis. The temperature observed during the experiment never overcame 35 °C. Although the lamp heat caused some evaporation/condensation of the solvent, the use of a closed container avoided loss of material and concentration changes. A control test was done with the system in the dark but heated at 35‒40 °C and no significant difference was observed in comparison with the non-heated one.

The Cr(VI) concentration was determined by applying the 1,5-diphenylcarbazide (DPC) method [50], in which the sample’s absorbance at 540 nm was measured with the UV–Vis spectrophotometer Cary 300 Scan (Varian, Palo Alto, USA). The concentration of HA in the samples was calculated using the UV absorbance at 254 nm [51,52,53,54,55], measured with the same equipment on samples before DPC addition. In the experiments with the effluent, the total Cr and Zn concentrations were measured by atomic absorption spectroscopy (AAS) using the spectrophotometer XplorAA (GBC Scientific Equipment, Braeside, Australia).

The Eh–pH (Pourbaix) diagram for the system Cr–H_2_O was generated using the HSC Chemistry 6.0 (Outokumpu, Helsinki, Finland) software. The stability areas of the Cr species were calculated considering the reduction potentials and equilibrium constants provided by the software at the temperature of 25 °C and for a total Cr(VI) concentration of 10 mg L^−1^.

## 3. Results and Discussion

### 3.1. Suspended Ce–ZrO_2_ Nanopowder

In previous works [22,23,24,25,26], the development and testing of Ce–ZrO_2_ nanoparticles was studied. An extensive characterization of the material was performed, including XRD, SEM, TEM, N_2_ adsorption/desorption, diffusive reflectance, and photo-electronic characterization. In summary, these analyses demonstrated that Ce–ZrO_2_, prepared by the same sol–gel procedure applied in the present work, presents both tetragonal and monoclinic phases with crystallite size around 15 nm [24]. The specific surface area of this material is 70 ± 7 m^2^ g^−1^, the total pore volume is 0.33 cm^3^.g^−1^, and pore sizes from 3 to 10 nm [25]. In spin trapping electron paramagnetic resonance (EPR) experiments, Ce-doped zirconia formed two paramagnetic species (trapped electrons as Zr^3+^ species and holes as O^−^ species, when irradiated by visible light (>420 nm) [56]. The formation of ^●^OH radicals under visible light irradiation using the spin trap DMPO (5,5-Dimethyl-1-Pyrroline-N-Oxide) [22] was also observed. These results confirmed the photoactivity of the material under visible light, and allowed its application in the photocatalytic degradation of 2-propanol [23], methylene blue [56], and humic acid [25].

In the following items, we will discuss the activity of Ce–ZrO2 nanopowder on photocatalytic reduction of Cr(VI) and the effects of the catalyst dosage and the initial pH on the Cr(VI) removal from model solutions containing only Cr(VI) or Cr(VI) + humic acid. 

#### 3.1.1. Effects of pH, HA, and Catalyst Dosage on Cr(VI) Adsorption 

**Figure 2:**
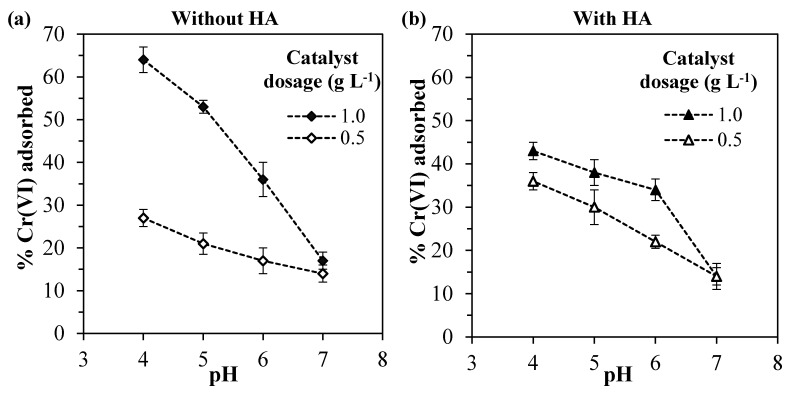
Percentage of Cr(VI) adsorbed for different pH values and catalyst dosages for the systems: (**a**) without humic acid and (**b**) with 10 mg L^−1^ of humic acid.

It can be observed in Figure 2 that the Cr(VI) adsorption increases with higher catalyst dosages and lower the pH values for the systems without and with HA.

Considering first the system without HA (Figure 2a), for pH values lower than 7, the use of double catalyst dosage led to an average increase of 2.3-fold in the amount of Cr(VI) adsorbed, since the higher mass of ZrO_2_ exposed more active sites available for adsorption [57]. The increase in the amount of Cr(VI) adsorbed with higher catalyst dosage was also reported by other authors, which also observed this non-linear behavior [58,59].

**Figure 3:**
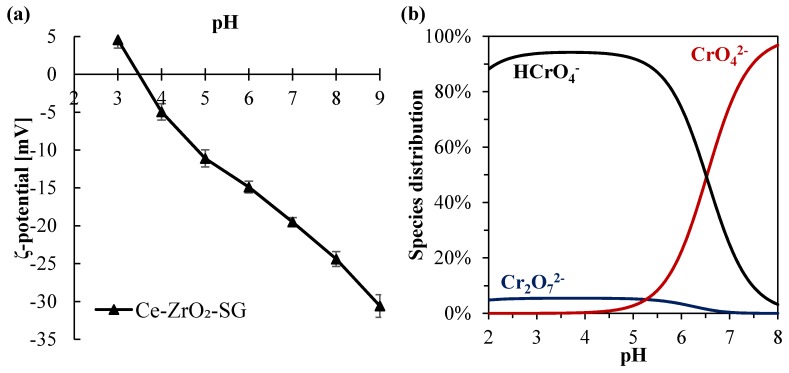
(**a**) Cr(VI) species distribution diagram for a total Cr(VI) concentration of 10 mg L^−1^ in water; (**b**) ζ-potential for Ce-doped zirconia.

Regarding the pH value, higher Cr(VI) amounts were adsorbed in more acidic environments. This can be explained by the reduction of the electrical repulsion between chromium species and the zirconia surface. In fact, analyzing the Cr(VI) species distribution (Figure 3b), it can be observed that for pH values lower than ~6.5, the prevalent chromium species changes from the bivalent anion CrO_4_^2–^ to the monovalent anion HCrO_4_^-^. At the same time, in a lower pH range, the surface charge of Ce–ZrO_2_ becomes less negative, as shown by ζ-potential measurements (Figure 3a), with the protonation of OH groups at the surface. Therefore, in more acid media, both Cr(VI) species and zirconia surface become less negatively charged, which reduces the repulsion and increases the adsorption, as reported by other authors for Cr(VI)–TiO_2_ systems [1,16].

Concerning the ζ-potential of Ce–ZrO_2_, this material exhibits a negative charge in a wide range of pH (>3.5) because of the presence of hydroxyl groups (ZrO_2_–OH) on zirconia surface [60]. Another author [61] also observed a high negative ζ-potential of ZrO_2_ as the pH increased.

Considering now the effect of humic acid, it is known that both Cr(VI) and HA compete for the adsorption at the zirconia surface [1] and that the number of active sites in the solid is limited. It should be noticed that in all the experiments, the adsorption of HA was greater than 80% (data not shown), which was favored by the range of pH utilized, since the humic acid adsorption is strongly increased in acid media [25,51,52,53,62,63].

Since, for the catalyst dosage of 1.0 g L^−1^, the presence of humic acid decreased the Cr(VI) adsorption, it may be concluded that HA is preferably adsorbed by zirconia, occupying the active sites of the catalyst. Nevertheless, for the catalyst dosage of 0.5 g L^–1^, the presence of HA has slightly increased the Cr(VI) adsorption. Since humic acid can form complexes with Cr(VI) species [64], these Cr(VI)–HA complexes may be adsorbed by the catalyst, increasing the Cr(VI) adsorption in comparison with the system without HA.

#### 3.1.2. Effects of HA and pH on the Cr(VI) Photocatalytic Reduction under Different Catalyst Dosages

**Figure 4:**
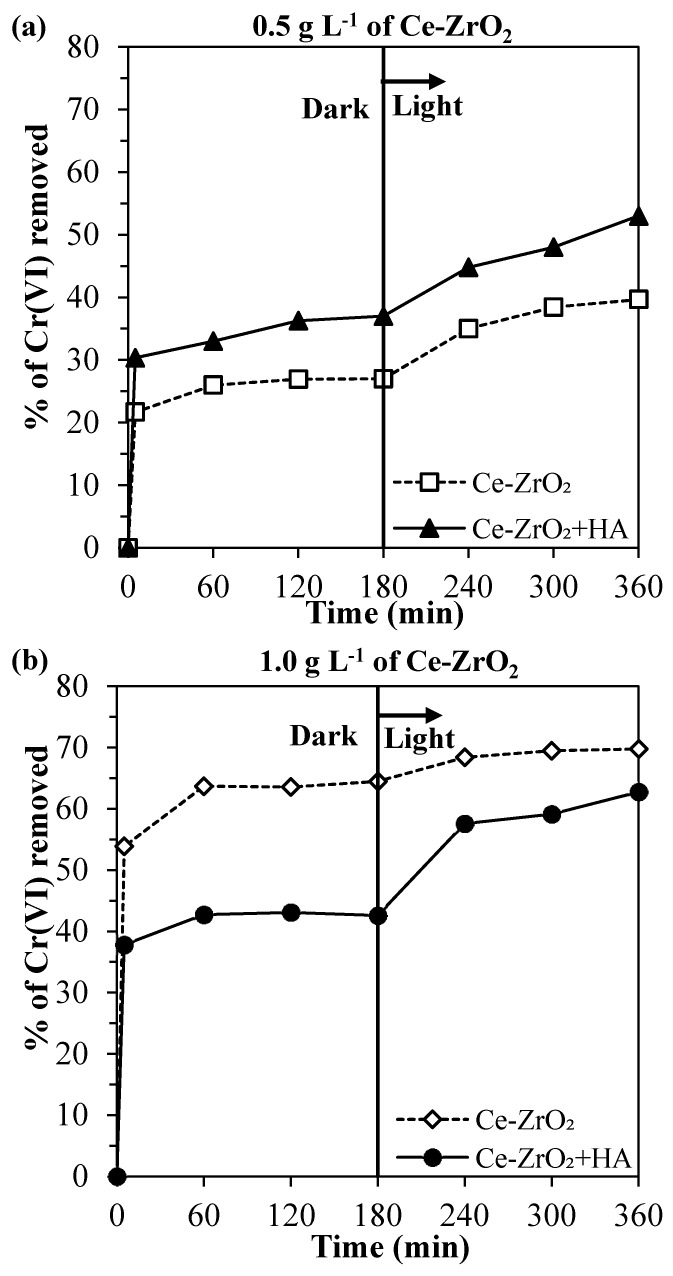
Cr(VI) removed at an initial pH of 4, without humic acid (HA) and with 10 mg L^−1^ of HA, in the experiments using: (**a**) 0.5 g L^−1^ of Ce–ZrO_2_ and (**b**) 1.0 g L^−1^ of Ce–ZrO_2_.

In Figure 4, it can be observed the total removal of Cr(VI), i.e., combined effects of adsorption in the dark and photocatalytic reduction. The adsorption was discussed in the previous section. Now, in order to understand the contribution of the Cr(VI) photocatalytic reduction and the effects of pH and HA, each parameter is going to be studied separately.

**Figure 5:**
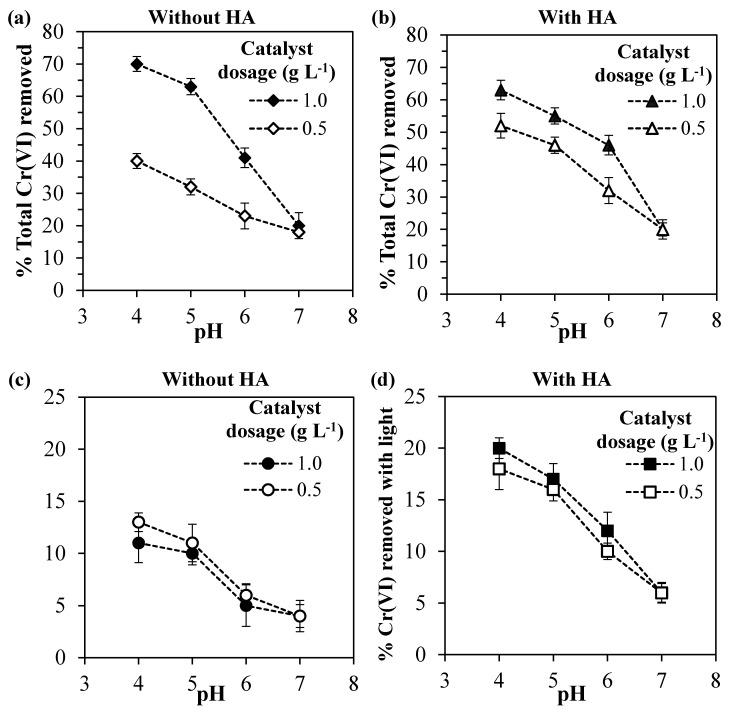
(**a,b**) Total Cr(VI) removed and (**c,d**) Cr(VI) removed by photoreduction for different pH values and catalyst dosages for the systems: (a,c) without humic acid and (b,d) with 10 mg L^−1^ of humic acid.

In the systems without humic acid (Figure 5a), as expected, the higher catalyst dosage led to the higher Cr(VI) removal, since the number of adsorbing sites and electrons generated in the conduction band increased with an increasing Ce–ZrO_2_ amount. However, the comparison with Figure 5c indicates that the photoreduced Cr(VI) amount does not increase significantly doubling the catalyst dosage, indicating that too much catalyst can shield the absorption of the incident light [1]. Further experiments are therefore needed to evaluate the optimal amount. 

The initial pH value has two effects on Cr(VI) removal. As discussed previously, acid media favor the Cr(VI) adsorption by reducing the electrical repulsion between Cr(VI) species and the Ce–ZrO_2_ surface. At the same time, also the photocatalytic reduction of Cr(VI) is favored at lower pH values, as shown in Figure 5c. 

**Figure 6:**
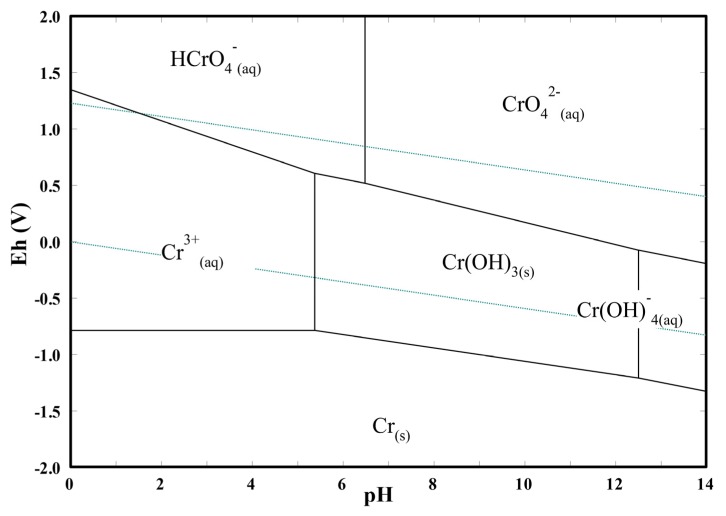
Potential in respect to the standard hydrogen electrode (Eh) versus pH diagram of Cr–H_2_O system at 25 °C for a total chromium concentration of 10 mg L^−1^.

There are many reasons explaining this phenomenon: 

1. The Cr(VI) photocatalytic reduction is controlled by a surface-reaction step [1,58,65]; thus, lowering the pH favors the Cr(VI) adsorption and then increases the Cr(VI) reduction;

2. As shown in Figure 6, the reduction potential of HCrO_4_^–^ (E^°^ = 1.35 V) is higher than that of CrO_4_^2–^ (E° = −0.13 V) [66]. Since at lower pH values HCrO_4_^–^ is the predominant species, the Cr(VI) reduction is favored;

3. The photocatalytic reduction of Cr(VI) is driven by the difference between the potential of the photo-induced electron at the catalyst CB and the reduction potential of Cr(VI) [1,15]. From Equations (1) and (2), it can be observed that the reduction potentials become more negative for lower pH values, while the potential of the photo-generated electron at CB shifts to more positive potentials [67], therefore the driving force for the Cr(VI) photocatalytic reduction increases in acid media.

HCrO_4_^–^ + 7H^+^ + 3e^–^ → Cr^3+^ + 4H_2_O (1)

CrO_4_^2–^ + 4H_2_O + 3e^–^ → Cr(OH)_3_ + 5OH^–^ (2)

In the presence of humic acid (Figure 5b), for the catalyst dosage of 0.5 g L^−1^, higher Cr(VI) total removal percentages were obtained in comparison with the systems without HA (Figure 5a), but for the catalyst dosage of 1.0 g L^−1^, the presence of HA did not change significantly the Cr(VI) total removal. The important effect of HA was to increase the percentages of the Cr(VI) removed by photocatalytic reduction (Figure 5c,d) for both catalyst dosages. In addition, when HA was present, the Cr(VI) photocatalytic reduction was less affected by the pH and the catalyst dosage. 

In order to explain these results, it should be considered that HA is involved in two different processes with opposite effects: 

1. HA is adsorbed at the photocatalyst surface, occupying the active sites for Cr(VI) adsorption and reduction, decreasing Cr(VI) removal efficiency; 

2. The adsorbed HA, being an electron-rich molecule, acts as a scavenger for positive holes at the ZrO_2_ valence band, which reduces the electron–hole recombination rate [59] and consequently increases the Cr(VI) photocatalytic reduction efficiency. This was also reported for Cr(VI) photocatalytic reduction using TiO_2_ in the presence of organic compounds, such as humic acid [1,16], phenol [16,68], ethanol [58,69], and citric acid [15]. 

Therefore, the final effect of HA on Cr(VI) removal is a balance between these two effects, which depends on the solid loading, pH, and HA concentration. For the zirconia loading of 0.5 g L^−1^, the HA increases both Cr(VI) adsorption and photocatalytic reduction, which leads to higher Cr(VI) total removals. For a loading of 1.0 g L^−1^, the Cr(VI) adsorption is hindered because HA is preferably adsorbed and occupies the active sites of the catalyst, but still, HA increases the Cr(VI) photocatalytic reduction by scavenging the holes on the VB. In agreement with these statements, the observed rate constants, considering a first-order reaction, were 0.11 and 0.16 h^−1^ for Cr(VI) and Cr(VI) + HA systems, respectively, using 1.0 g L^−1^ of the catalyst. It is worth noting that in all the tests the removal of HA after the end of the irradiation was higher than 90% (data not shown). 

**Figure 7:**
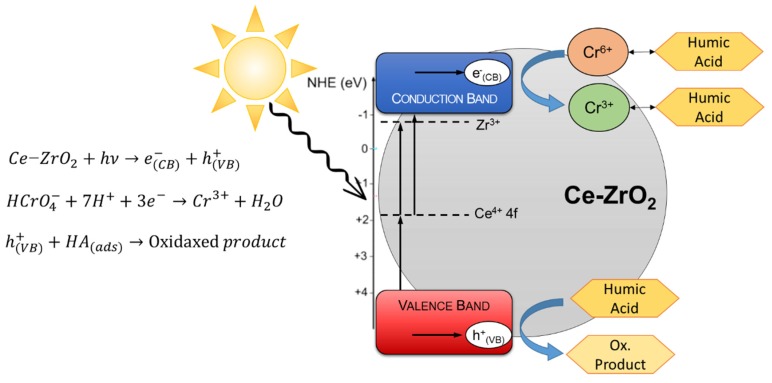
Proposed mechanism for the photocatalytic reduction of Cr(VI) in the presence of HA using Ce–ZrO_2_ under visible light irradiation.

In view of the experimental results and previous works with zirconia [22,25,56] and Cr(VI)+TiO_2_ works [1,4,68], a simplified mechanism was proposed in Figure 7. 

In order to evaluate the fate of the adsorbed Cr species on Ce–ZrO_2_, the catalysts used for the experiments shown in Figure 4b (1 g L^−1^ of Ce–ZrO_2_ and initial pH of four) were recovered and washed with strong basic solutions to promote the complete desorption. Then, the washing solutions were analyzed in order to determine the amount of Cr(VI) released. Once knowing the initial and final Cr(VI) concentrations, and the amount that was adsorbed, it was calculated, through a mass balance, how much Cr(VI) was reduced to Cr(III). As a result, it was observed that ~60% of the adsorbed chromium was reduced to Cr(III) and the rest remained as Cr(VI). For the non-adsorbed Cr(VI), which remained in the starting solution, 36% was reduced to Cr(III) in the presence of HA, whereas this value dropped to 25% without HA. This result corroborates with the proposed mechanism, in which the photocatalytic reduction depends on the adsorption of the Cr(VI) species at the catalyst surface and that the HA favors the photoreduction by acting as a hole scavenger. In addition, the presence of HA can be considered beneficial since, in its presence, Cr(III)–HA complexes are formed when Cr(VI) is reduced to Cr(III). These complexes are less toxic than Cr(III) species [32]. 

Another positive effect of HA reported in the literature is the direct electron transfer from the light-excited HA to Cr(VI), promoting the metal reduction, which would require lower energies than the semiconductor bandgap [59]. However, when experiments without Ce–ZrO_2_, only with Cr(VI) and humic acid, were performed, very low Cr(VI) removals were observed, which confirms the actual photocatalytic activity of Ce–ZrO_2_.

### 3.2. Immobilized Ce–ZrO_2_

**Figure 8:**
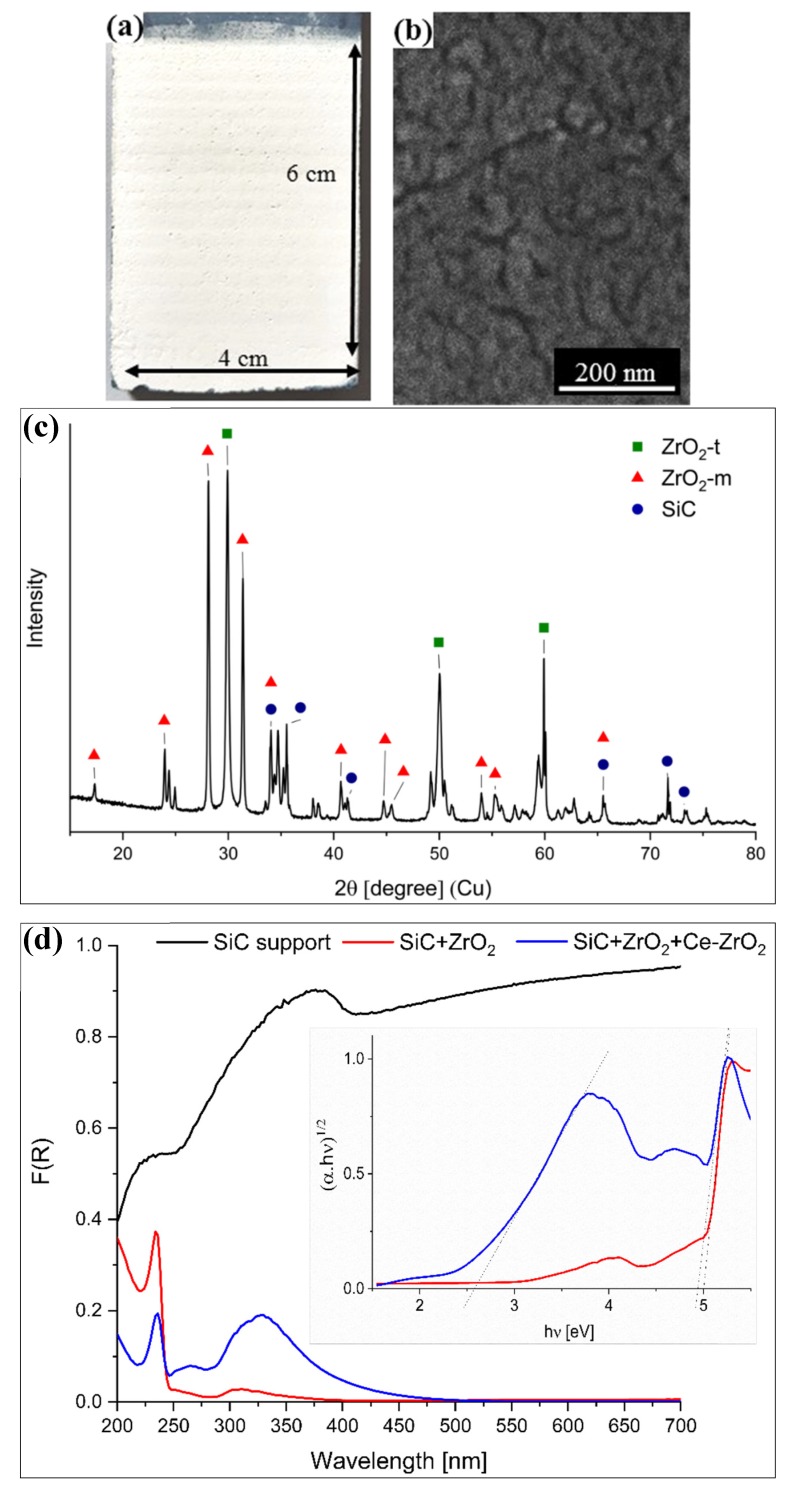
(**a**) Photo, (**b**) FE-–EM image, and (**c**) X-ray diffractogram of the immobilized Ce–ZrO_2_ on the silicon carbide (SiC) support. (**d**) Absorbance spectra of the samples obtained by applying the Kubelka–Munk function, F(R), to the diffuse reflectance spectra. The inset is the Tauc plot of the SiC support + ZrO_2_ intermediate layer and the immobilized Ce–ZrO_2_.

In order to increase the photocatalytic reduction efficiency and avoid the use of suspended nanoparticles, hard to recover, Ce–ZrO_2_ was immobilized on a silicon carbide support coated with ZrO_2_ as the intermediate layer (Figure 8a). The FE–SEM analysis of the Ce–ZrO_2_ immobilized on the support (Figure 8b) indicated the presence of nanometric particles and a layer thickness of around 2 µm. Previous TEM analysis confirmed that Ce–ZrO_2_ nanoparticles are a mix of nanometric grains of about 10 nm with twinned grains of a relatively larger dimension (from 15 to 60 nm) and some bigger crystals of about 100 nm [26]. 

In the X-ray diffractogram of the immobilized Ce–ZrO_2_ (Figure 8c), it is possible to identify a SiC phase (ICDD Ref. code 00-049-1428), which corresponds to the silicon carbide support. The zirconia phases observed correspond to the ZrO_2_ intermediate layer and to the immobilized Ce–ZrO_2_. As obtained from previous XRD analysis of the unsupported Ce-doped zirconia [25], both monoclinic (ZrO_2_-m, ICSD #658755) and tetragonal (ZrO_2_-t, ICSD #66781) phases form during the synthesis. No evidence related to a preferred phase for the photocatalytic activity were reported; however, the presence of both phases could enhance the charge separation at the interface between the two polymorphs [26] reducing the charge recombination probability, similarly to what proposed for anatase and rutile in TiO_2_ P25 (Evonik) [70]. 

Figure 8d presents the UV–Vis absorption spectra obtained by Diffuse Reflectance Spectroscopy (DRS) of the SiC support, the SiC support with the ZrO_2_ intermediate layer and the immobilized Ce–ZrO_2_. Since the silicon carbide support is black, it absorbs light in the whole UV–Vis spectra, whereas when coated with the white ZrO_2_ intermediate layer, only UV light is absorbed. The spectrum obtained for ZrO_2_ layer matches that reported for pristine ZrO_2_ [22,23,24], in which the bandgap transition occurring at about 250 nm (5 eV) is due to the excitation of the electrons from the VB to the CB of this oxide. A weak absorption between 250 nm and 350 nm is caused by some point defects present in the material [71]. In the spectrum of the Ce–ZrO_2_ immobilized on the SiC support with the ZrO_2_ intermediate layer, an absorption shoulder centered at ca. 330 nm with a tail in the visible region is observed. Analyzing the bandgap transitions, two E_gap_ values are reported, 4.9 and 2.6 eV. The first value is associated with the fundamental VB → CB transition of ZrO_2_, which was practically unaffected by the Ce doping, the second value is due to the absorption band associated to the VB → Ce 4f charge transfer transition [23]: this result indicates that the immobilized Ce-doped zirconia has the potential to work as a photocatalyst under visible light irradiation.

#### 3.2.1. Experiments Using a Solution Containing Cr(VI)

**Figure 9:**
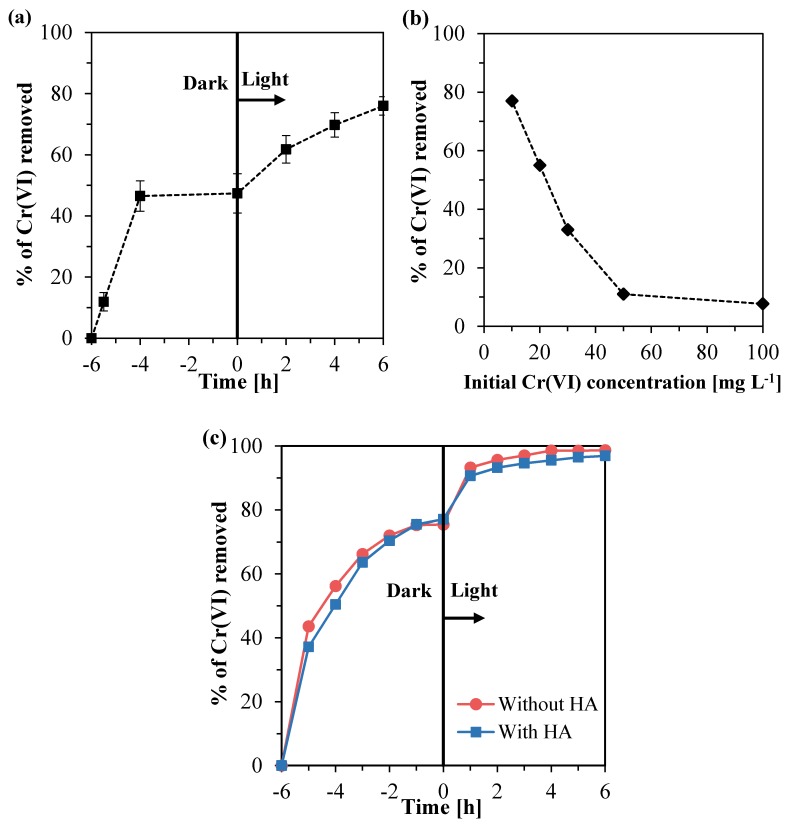
Percentages of Cr(VI) removed using immobilized Ce–ZrO_2_ in the tests with: (**a**) model solution containing 10 mg L^−1^ of Cr(VI); (**b**) model solutions containing different initial Cr(VI) concentrations; (**c**) diluted galvanizing industry effluent.

In Figure 9a, it is shown that, using the immobilized Ce–ZrO₂ on the silicon carbide support, the removal of Cr(VI) reached 77% for a model solution containing 10 mg L^−1^ of Cr(VI), no humic acid, and at an initial pH of four. It can be observed that a strong adsorption of Cr(VI) occurred in the dark period, as reported for the suspended catalyst. However, a direct comparison with the powdery catalyst is not possible since it is difficult to determine the amount of Ce–ZrO₂ that was immobilized on the support and the related active fraction. Nevertheless, when the visible light irradiation started, it was clearly observed that the photocatalytic reduction of Cr(VI) occurred. Considering a first-order reaction, the observed rate constant (K_obs_) was 0.13 h^−1^, value compatible with the ones obtained in the presence of a suspended catalyst.

The effect of the initial Cr(VI) concentration on the Cr(VI) removal with immobilized Ce–ZrO_2_ at pH 4 is shown in Figure 9b. It can be observed that for an initial Cr(VI) concentration of 10 mg L^−1^, high removal of chromium was achieved, whereas, for the concentrations of 50 and 100 mg L^−1^, the Cr(IV) removal did not overcome the values around 11%. Thus, it can be concluded that the removal of Cr(VI) is significantly dependent on the initial Cr(VI) concentration in the feed and follows an inverse relationship with the concentration, as reported by other authors [4,72,73]. The main reason for that is that increasing the concentration of Cr(VI) more light is absorbed by the solution, and therefore, fewer photons reach the catalyst surface to promote the charge separation and consequently the Cr(VI) reduction [4].

#### 3.2.2. Experiments with the Galvanizing Industry Effluent

In view of the satisfactory results obtained with the supported Ce–ZrO_2_ for the model Cr(VI) solution, the next step was to evaluate the efficiency of this material in the treatment of a real Cr(VI) effluent, since complex matrixes can affect significantly the photocatalytic mechanism [74,75].

The galvanizing industry effluent studied here contains a high concentration of Zn and Cr(VI), 1789 and 77 mg L^−1^, respectively. As reported in the previous item, the Cr(VI) removal efficiency decreases considerably at higher Cr(VI) concentrations. Therefore, prior to the experiments, the effluent was diluted seven times in order to achieve a Cr(VI) concentration of 11 mg L^−1^. It is worth noting that the total Cr concentration in the effluent is 89 mg L^−1^, which means that a small amount of dissolved Cr(III) is also present. Since the effluent does not contain dissolved organic matter, one test was made with the diluted effluent spiked with HA. The results of the photocatalytic reduction of Cr(VI) using immobilized Ce–ZrO_2_ are shown in Table 1.

From Figure 9c, it is possible to observe that for the diluted effluent, the adsorption of Cr(VI) is slower than for the model solution (Figure 9a). This may be caused by the high amount of Zn^2+^ cations present in the solution, which also competes for adsorption on the negatively charged Ce–ZrO_2_ surface. As shown in Table 2, the variations in Zn concentration due to its adsorption are four times greater than the ones obtained for Cr(VI). From this table, it can also be seen that 4% to 5% of the zinc was removed after light irradiation. However, there is no evidence to confirm that this zinc removal was due to adsorption or photocatalytic reduction, since the standard reduction potential of Zn^2+^ (−0.76 V) is much lower than the one of Cr(VI) [66].

The Cr(VI) removal efficiencies for the model Cr(VI) solution and the diluted galvanizing effluent were ~76% and >97%, respectively. The first reason for the higher removal of Cr(VI) with the galvanizing effluent is that the effluent pH value is lower (~3.5) than the one tested for the model solution (4.0). As discussed previously, the Cr(VI) photoreduction is strongly favored in more acidic media. Another reason for the higher removal of Cr(VI) is the presence of Cr(III) and Zn(II) in the galvanizing effluent, which help to maintain pH stability by forming hydroxo-complexes acting as pH buffer [72]. 

On the contrary, the presence of HA does not seem to have a significant effect on Cr(VI) removal, even if the effect was clearly visible when the suspended catalyst powder was used. Actually, in the case of the diluted effluent spiked with HA, the humic acid was no longer detectable by the UV–Vis analyses carried out after a 1 h experiment in the dark, as it was completely adsorbed by the immobilized Ce–ZrO_2_ and also by the mesoporous silicon carbide support. Therefore, there was no humic acid available anymore to participate in the reaction and affect the Cr(VI) removal. 

The final concentrations of Cr(VI) in the samples (Table 2) indicate that the proposed process is suitable for the treatment of the galvanizing industry effluent, since more than 97% of chromium was removed. The limit of discharge of Cr(VI) species for industrial waste streams to be discharged to surface water varies by country, but in general, this limit is in the range of 0.1 and 0.5 mg L^−1^ [76,77]. Therefore, the diluted effluent after photocatalytic treatment with immobilized Ce–ZrO2 would be within the discharge limit for Cr(VI).

## 4. Conclusions

In the present work, we studied the removal of Cr(VI) by photocatalytic reduction promoted by suspended and immobilized Ce–ZrO_2_ under visible light irradiation. For the model Cr(VI) solution, it was observed that higher catalyst dosages and lower initial pH values favor Cr(VI) adsorption and photoreduction. The reason is that in more acidic media, the electrostatic repulsion between the zirconia surface and Cr species is limited. At the same time, the driving force for the reaction is increased because CrO_4_^2–^ converts to HCrO_4_^-^, which has a higher reduction potential and less repulsion by the zirconia surface.

Regarding the effects of humic acid, there is a balance between beneficial and detrimental effects. Since HA is preferably adsorbed by Ce–ZrO_2_, it reduces Cr(VI) adsorption by occupying the active sites. On the other hand, HA acts as a hole scavenger, reducing the electron–hole recombination, favoring then the photocatalytic reduction of Cr(VI). Therefore, the combination of these effects on the total Cr(VI) removal was dependent on the catalyst dosage, but in all cases, the amounts of Cr(VI) reduced with the irradiation were higher in the presence of HA, even at higher pH values. In the best condition, it was possible to remove 70% of Cr(VI) from the model solution containing only Cr(VI) at an initial pH of four, using 1.0 g L^−1^ of Ce–ZrO_2_. 

The experiments with the supported Ce–ZrO_2_ demonstrated the feasibility of immobilizing this catalyst without changing its photocatalytic properties. The immobilized Ce–ZrO_2_ was able to treat a diluted galvanizing industry effluent with a high concentration of zinc, achieving Cr(VI) removals greater than 97% after visible light irradiation, which would allow its discharge into surface waters. The presence of HA did not affect the processes.

These results indicate that immobilized Ce-doped zirconia can be applied to treat Cr(VI) effluents even in the presence of other metals and naturally occurring organic matter, such as humic-like substances. The advantages of this material are its great chemical and mechanical resistances and the chance to avoid nanoparticle recovery. In addition, there is the possibility to use visible light sources, such as solar light, which contributes to the development of more sustainable, cleaner, and cost-effective wastewater treatments.

## Figures and Tables

**Table 1:** Removals of total Cr, Cr(VI), and Zn for the diluted effluent treatment with the immobilized Ce–ZrO_2_.

	Without HA		With HA (10 mg L^−1^)	
	After 6 h in Dark	After 6 h of Irradiation	After 6 h in Dark	After 6 h of Irradiation
**Removal of Cr total [%]**	77.1	98.4	75.5	98.8
**Removal of Cr(VI) [%]**	77.1	97.2	75.5	97.3
**Removal of Zn [%]**	13.6	19.9	12.6	17.6

**Table 2:** Concentrations of zinc in samples from the experiments with the diluted galvanizing effluent with and without humic acid.

	Without HA		With HA (10 mg L^−1^)	
	Cr(VI)[mg L^–1^]	Zn[mg L^–1^]	Cr(VI)[mg L^–1^]	Zn[mg L^–1^]
**Initial**	11	254	11	254
**After 6 h in Dark**	2.5	219	2.2	222
**After 6 h of Irradiation**	0.1	205	0.3	211
